# Petroleum pipeline monitoring using an internet of things (IoT) platform

**DOI:** 10.1007/s42452-021-04225-z

**Published:** 2021-01-23

**Authors:** E. N. Aba, O. A. Olugboji, A. Nasir, M. A. Olutoye, O. Adedipe

**Affiliations:** 1 Department of Mechanical Engineering, Federal University of Technology, Minna, Nigeria; 2 Department of Chemical Engineering, Federal University of Technology, Minna, Nigeria

**Keywords:** Internet of things, Sensors, Pipeline monitoring, ThingSpeak

## Abstract

In this study, we present the use of an internet of things (IoT) analytics platform service to mimic real-time pipeline monitoring and determine the location of damage on a pipeline. Pressure pulses, based on the principle of vibration in pipes are used for pipeline monitoring in this study. The principle of time delay between pulse arrivals at sensor positions is also adopted in this study. An Arduino and a Wi-Fi module were combined, programmed and used to produce a wireless communication device which communicates with the ThingSpeak internet of things (IoT) analytics platform. A total of five channels were created on the platform to collect data from the five sensors that were used in the experimental test rig that made use of wireless communication device. Signal data was collected once every 15 s and all the channels were updated every 2 min. ThingSpeak provided instant visualizations of data posted by the wireless communication device. Online analysis and processing of the data was performed as it came in. A second test rig was built that made use of a data logger for processing of data. The measured velocity of pulse propagation using the data logger and air as transport fluid was 355 m/s. The computed estimates of event location for the 50 measurements taken ranged between 4.243 m and 4.246 m. This had a scatter of just 3 mm against the actual measured event location of 4.23 m. The experimental results obtained showed that the performance of the wireless communication device compared satisfactorily with the data logger and is capable of detecting the location of damage on real pipelines when used for real time monitoring.

Using this communication device and an analytics platform, real-time monitoring of pipelines can be carried out from any location in the world on any internet-enabled device.

## Introduction

Pipelines are considered as the major globally recognised means of transporting petroleum products. These pipelines are usually damaged as a result of natural events (erosion, earthquakes, etc.) or due to third party activities (explosions, drilling activities, vehicular movement, etc.). Methods of monitoring of pipelines include intermittent appraisal of pipelines, use of pipeline integrity management systems, on-the-ground and air surveillance of pipelines by security forces. High cost, planning complexity, and lack of proper access routes are major drawbacks for these monitoring methods. A great challenge that pipeline operators have faced in the past has been that of real-time monitoring of pipelines that is not restricted to control rooms in a particular location. Previous research works on pipeline monitoring have rarely focused on real time transmission and monitoring of damage data wirelessly to an Internet-of-Things (IoT) platform. Previous research works have focused on the design/development of intelligence pipeline inspection gauges (pigs) to achieve this [1]. Available in the world today is about 2.5 million kilometres of hydrocarbon pipeline [2]. That is enough to go around the earth’s circumference at least 62 times because the earth has a circumference of about 40,000 km [3]. For different reasons, a good percentage of pipelines across the world are considered impossible to pig. These reasons include:

- Tight twists in the line do not permit the inflexible exoskeleton of the pig to go through
- Blockages brought about by silt and pollutants may go about as hindrances to the way of a pig [4].
- Pipes may have various widths which forbid the section of these torpedo-like structures [4].
- There are valves in the pipeline that forever discourage the entry of anything besides a gas or liquid [4].
- There might be no immediate passage into a line [4].

The process of pigging is a very expensive one. Estimates have shown that pipeline monitoring and inspection by pigs can cost as much as $56,000 per km of pipeline [4]. Taking into consideration that 25 percent of the world’s pipelines fall into the ‘un-piggable’ class, it can be estimated that companies are spending close to $105 billion on pigging the world’s hydrocarbon pipelines [4]. This is more than the annual gross domestic product of a lot of countries. Creating a pigging system for pipeline inspection and monitoring is a very messy and labor intensive process. A very demanding planning process is required to make sure that operations are maintained within Health, Safety and Environment (HSE) parameters because most pigging processes run whilst pipelines are in service. Trained crew may require hours to correctly load the pig into the pipe after the planning is complete; and the running distance will only stretch to a handful of kilometers. As a result of this, depending on the pipe, before an inspection pattern can emerge, pigs may need to be launched severally. Smart pigs use high-tech innovation such as transmitters, sensors, GPS, eddy current, magnetic fields, ultrasonic and acoustics to distinguish and analyze potential issues [5].

The use of an Arduino, Wi-Fi module and the ThingSpeak IoT platform is advocated for to achieve real time monitoring of a pipeline from anywhere in the world.

Most times, damages caused by impulsive events generate a pressure pulse that propagates in both directions through the fluid in the pipe. Detection and measurement of these pressure pulses can be carried out at points remote from the event. These measured pulses contain information about the event and can be used to monitor these pipelines to detect and locate damages along the pipeline.

Methods of pipeline line monitoring from previous research exists. These methods have their various merits and demerits. The negative pressure wave (NPW) method of pipeline monitoring was used by Junxiao et al. [6] in carrying out damage detection in a gas pipeline. A stress wave generated by leakage in the pipeline, which propagates along the pipeline from the leakage point to both ends was used. Also used was the hoop strain variation along the pipe leakage point to both ends, and the hoop strain variation along the pipe wall. The pipeline monitoring method of wavelet packet-based damage index matrix was developed by Guofeng et al. [7] and was used to identify the crack damage in pipeline structure using a stress wave propagation approach with piezo-ceramic transducers. In the work, four cracks were artificially cut on the specimen, and each crack had six damage cases corresponding to different crack depths. This aided them to simulate cracks at different locations with different damage degrees. In each damage case, they used one piezo-ceramic transducer as an actuator to generate a stress wave to propagate along the pipeline specimen, and the other piezo-ceramic transducers were used as sensors to detect the wave responses. Golmohamadi [8] used the pipeline monitoring method of wavelet transform for processing signals to recognize damage and leak location in a hardware-based technique which used ultrasonic wave emission. The pipeline monitoring method of vibrothermography was adopted by Changhang et al. [9]. A low-power piezo-ceramic transducer was used as the actuator of vibrothermography. Its ability to detect multiple surface cracks in a metal part was explored. Also, the Fourier transform signal processing technique was employed in the work and results showed that all cracks can be detected conveniently and simultaneously by using the proposed low-power vibrothermography. Enrique et al. [10] combined the guided waves and electro-mechanical impedance techniques based on smart sensing to define a new and integrated damage detection procedure. This combination of techniques was studied by them and they proposed a new integrated damage indicator based on Electro-Mechanical Power Dissipation (EMPD). They tested the applicability of their proposed technique through different experimental tests, with both lab-scale and real-scale structures. A new method of pipeline monitoring was proposed by Kia et al. [11] as damage detection of a concrete column structure, subjected to blast loads using embedded piezo-ceramic smart aggregates (SAs) was investigated. The work proposed an active-sensing based approach in which the embedded SAs act as actuators and sensors that can respectively generate and detect stress waves.

Vibration-based methods for pipeline monitoring have been found to be very effective. The method of time delay between pressure pulse arrivals was found to be very effective in the location of leaks on a pipeline [12]. Due to unwanted interference noise from traffic, water, wind and other sources, the acoustic method of pipeline monitoring was found to be inefficient in the determination of a leak in a pipe [13]. Inaccurate modelling of the transients and boundary conditions in a pipe network was found to be a major drawback of the inverse least square method of pipeline monitoring [14].

The oil and gas sector has been slow to embrace IoT technology despite having pipelines and refining facilities, and instrumentation on drilling rigs for decades. Only recently has the extraction industry begun to work with modern IoT [15]. This change, is in part due to energy prices that have taken a hit in recent times; and most especially due to the coronavirus pandemic. There is an urgent need for oil-producing nations to safeguard their oil pipeline facilities from oil saboteurs in a bid to save their earnings from oil and gas production. This will in no small measure contribute to the funding of their national budget and boost their foreign exchange earnings [16]. Oil companies have been working to cut costs; and integration and automation is one of the easiest places to achieve this. An IoT solution that would be able to tie all these different threads of data together has now become a viable option for petroleum companies seeking to minimize human error and obtain real-time insights from the wide range of instrumentation present on the average petroleum pipeline [15].

It was referenced in [17] that coordinating IoT with contributions from specialists who can get to the live information distantly and give input by means of a few video real time channels would permit pipeline administrators access the perfect data at the perfect time, with the best examination which would empower them to move from a reactive to a proactive/prescient operational point of view.

IoT can play several roles in monitoring of pipelines. Operational data from electric submersible pump can be monitored to detect potential failure and automatically stop the pump to prevent damage, and in-turn notify operators to repair or replace the pump based on current machine and maintenance models [18]. IoT can also be used in pipeline optimization, where it can shut down a valve and send an alert to a mobile device to avoid a major disruption or damage a pipeline [18].

The upsides of embracing IoT for pipeline monitoring are various. Joshi [19] expressed that without IoT, organizations would need to depend on people to do routine checks and support. The IoT framework assists with chopping down manual checks because of its capacity to screen pipelines continuously. The information acquired progressively can be utilized to diminish significant perils that are connected with pipeline spillages and other undesirable circumstances. Another bit of leeway of utilizing IoT in pipeline observing as expressed in [19] is the productive administration of workers. The requirement for intermittent human assessment and human resource is significantly diminished as representatives would possibly be needed to complete support when a variation from the norm is identified.

To the best of the author’s knowledge, not much works exits in the use of IoT for monitoring of petroleum pipelines but related works to the scope of this study exists. Notably, Cheddadi et al. [20] proposed a practical IoT framework to assemble and screen continuously electric and environmental information of a PV solar station. In the work, a low cost data pipeline for observing the ecological and electrical boundaries in a photovoltaic station was planned to gather, cycle, store, and dissect information. The ESP32 DEVKIT V1 was utilized as the microcontroller in the proposed observing framework for assortment and handling of approaching information from sensors prior to communicating the prepared information to the cloud through implicit Wi-Fi.

Pipeline monitoring and inspection technologies have garnered significant attention across the globe. In this study, sensor networks were used to determine the location of bursts, leaks and other anomalies (damages) in general pipeline systems using pressure pulses based on the principle of vibration in pipes. The principle of time delay between pulse arrivals at sensor positions was adopted in this study. The competitive advantage of this research work and its contribution to knowledge lies in its ability to perform real-time damage location through the use of a combination of wave propagation, an active sensor network, a wireless data transmission system, and an Internet of Thing (IoT) platform. With this system, the transmission of damage data captured by sensors to the monitoring room is achieved wirelessly, thereby making the monitoring of pipelines in real-time from any location in the world possible.

To the best of the authors knowledge, previous research works on pipeline monitoring have rarely focused on real time transmission and monitoring of damage data wirelessly to an Internet-of-Things (IoT) platform. Additionally, the investigators utilized an Arduino and Wi-fi-module to produce a wireless communication device to capture pressure pulses from sensors on pipes on an experimental test rig and transmit these pulse data wirelessly to the ThingSpeak analytics platform. The following section discusses the data and methods utilized for this study. The third section discusses the findings based on methods adopted. The fourth section outlines the conclusions drawn from this study.

## Materials and methods

### Materials

The major materials that were used for this work are:

1. Flexible polyethylene hose pipe
2. A test rig

The experimental setups for this work are shown in Figs. 4 and 5. The major equipment for the research, and a brief about their use is presented thus:

- TCAM piezoelectric sensors (diameter 15 cm; thickness 0.35 mm; Model number: 8QQ0302) for detection of propagated pulses along pipeline.
- Pulse generator for generation of sharp fronted pressure pulses in the pipe.
- Pico Log 1012 (10 bits, 12 channels data acquisition module with serial number pl1000.en r2 10.05.2013) for recording and processing of signals from pressure pulses at specified sampling rate
- Wireless communication device for processing and transmission of signal data received from sensors wirelessly to the ThingSpeak IoT analytics platform.
- Laptop computer: Connected to data acquisition module for processing of and visualisation of data.

#### Wireless communication device

A wireless communication device was developed to carry out wireless transmission of signal data from the sensors to the ThingSpeak IoT analytics platform. The device consisted of an Arduino and a Wi-Fi module which were integrated with the sensors that were placed on the pipelines. The Arduino was programmed to communicate with the ThingSpeak IoT analytics platform thereby sending signal data to the platform for visualization and analysis.

#### Arduino UNO R3

An Arduino is an open-source electronics platform based on easy-to-use hardware and software. Inputs like light on a sensor, a finger on a button, or a Twitter message can be read by an Arduino board. These inputs are then turned it into an output like activating a motor, turning on an LED, publishing something online [21]. The Hwayeh CH34og + MEGA 328P Arduino was used in the development of the wireless communication device. It made use of an Atmel 328 microprocessor controller. The Arduino was programmed to collect data once every 15 s and update the channels on the analytics platform once every 2 min. The Arduino code is called a ‘sketch’ which is a short program that is run over and over by the device.

#### Wi-Fi module

The ESP01 ESP8266 Wi-Fi module was used for developing the wireless communication device. ESP8266 is a standalone transceiver that is of minimal cost which can be adopted in an IoT structure. This Wi-Fi module enables internet connection to sensors and other application specific devices through its general purpose input/output (GPIO) pins. It is very user friendly with minimal development up-front, and minimal loading during runtime. It is designed to occupy minimal printed circuit board (PCB) area and connects with the server by means of the TCP or UDP communication protocol [22].

### Methods

#### Petroleum pipeline monitoring based on pressure pulse analysis

A pressure pulse is generated when a pipe is damaged and this pressure pulse propagates in both directions away from the location of the damage. Both pressure pulses are eventually reflected when they reach the boundaries of the pipelines. This work looked at the monitoring of vibration-based events on a pipeline considering a situation with air flowing through the pipe. This required the use of sensors on opposite sides of the event. Using a pressure measurement sampled at a high frequency at several points along the pipe, the travel times of the pressure pulses can be found.

#### Propagation of damage-induced pressure pulses in an air-filled pipe

**Fig. 1:**
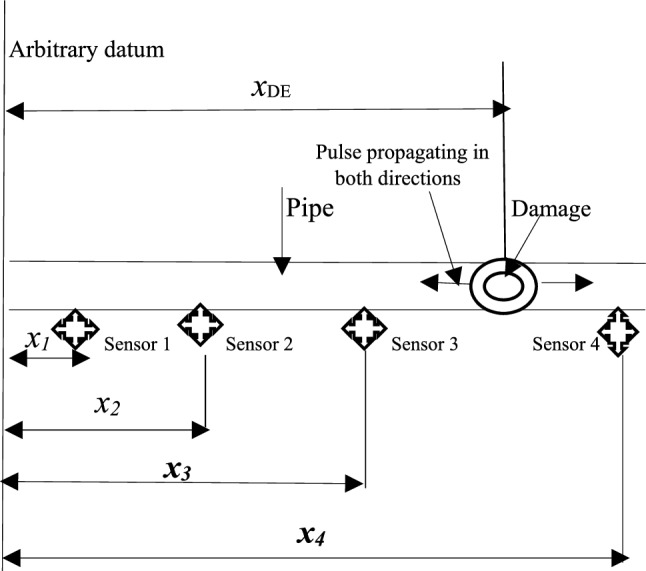
Schematic representation of sensors on a pipeline

The pulse arrival times in a pipe and the sensor positions along the pipe can be used to monitor a pipe and also determine the location of an event along a pipe. These events could either be caused by drilling, impact, explosion, etc. In Fig. 1, the schematic representation of a pipeline with four sensors placed along it is shown. These sensors are denoted by 1, 2, 3, 4, and are at distances *x*_1_, *x*_2_, *x*_3_, and *x*_4_ from some boundary. The arrival times of some generated pulses caused by a damage-inducing impulsive event occurring at an unknown location is recorded as *t*_1_, *t*_2_, *t*_3_, and *t*_4_ by the four sensors. Sensor 3 or 4 may be used to determine the location of the event on the pipe shown in Fig. 1. The occurrence of a damage event on a pipe leads to a change in pressure within the pipe which eventually leads to the generation of pressure pulses. Every increase or decrease in pressure travels at a velocity *C*_*p*_ in the form of a pressure pulse through the fluid filled pipe in both directions.

This pulse propagation velocity can be measured from the arrival of pulses at the same side of the event which are sensors 3 and 2, or sensors 2 and 1 as in the case shown in Fig. 1. Thus, the exact location of the damage event from sensor 3 is calculated by:

1 $$ x_{DE3} = \frac{{\left( {{ }t_{34} { }C_{p} { } + x_{43} } \right)}}{2} $$

or from sensor 4,

2 $$ x_{DE4} = \frac{{\left( {x_{43} - C_{p} t_{34} } \right)}}{2} $$

where $x_{43}$ = distance between sensors 4 and 3.

$t_{34}$ = time delay between arrival times at two selected sensors.

$C_{p}$ = pressure pulse velocity.

To determine the arrival times of signals at each sensor, the cross correlation technique was used.

#### ThingSpeak IoT analytics platform

ThingSpeak is an IoT platform service that enables live data streams to be viewed and analyzed from sensor devices in the cloud [23]. This platform enables you to perform data analysis on data collected from remote devices with Matlab® codes in real-time. You can sign up and create channels on the platform. These channels are configured via written codes to communicate with the desired sensors.

**Fig. 2:**
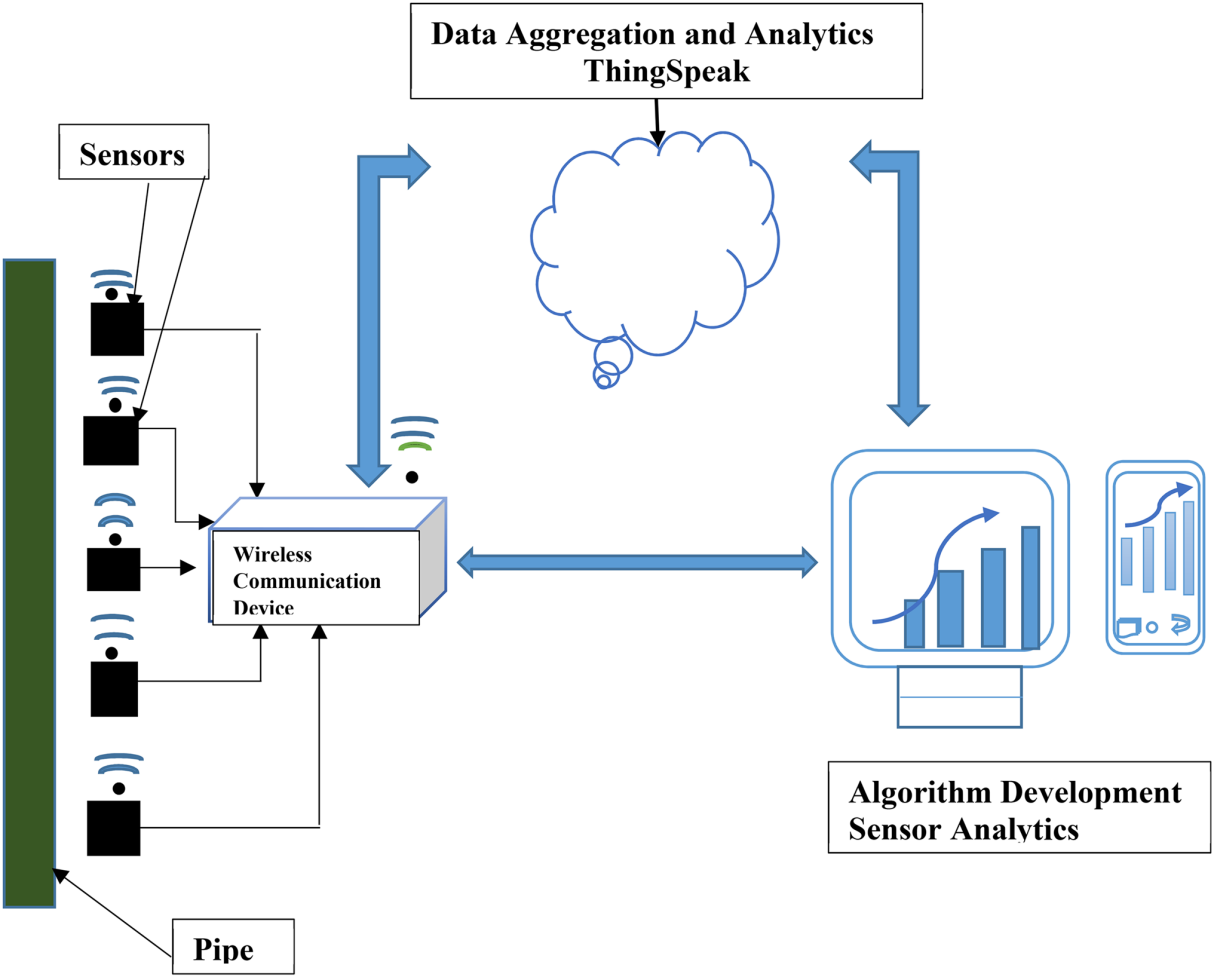
IoT system adopted for pipeline monitoring

A total of five channels were created on the ThingSpeak platform to collect data from the five sensors that were used in the experimental test rig. Signal data was collected once every 15 s and all the channels were updated every 2 min. ThingSpeak provided instant visualizations of data posted by the wireless communication devices. Online analysis and processing of the data was performed as it came in. Figure 2 illustrates the Internet of Things process that was carried out in this work.

The right side of the diagram depicts the algorithm development associated with the IoT application. Here insight was made into the collected data by performing historical analysis on the data.

#### Experimental validation

**Fig. 3:**
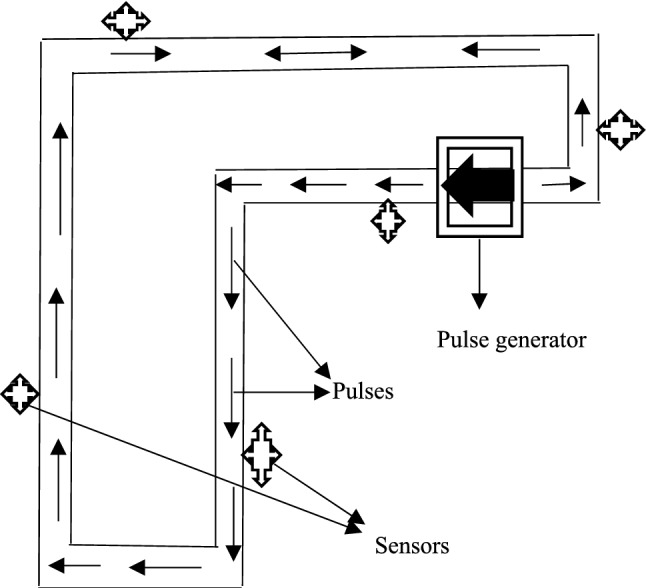
Schematic of experimental flow loop

**Fig. 4:**
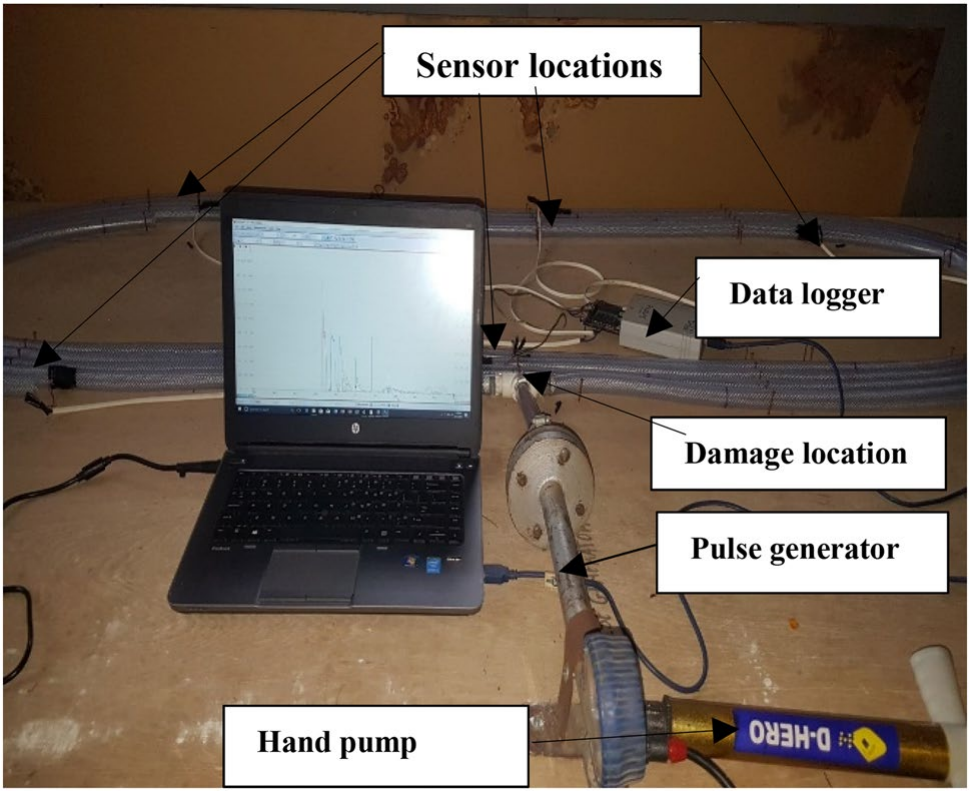
Experimental test rig1 showing various components of the rig with Pico Log as the data capturing device [25]

**Fig. 5:**
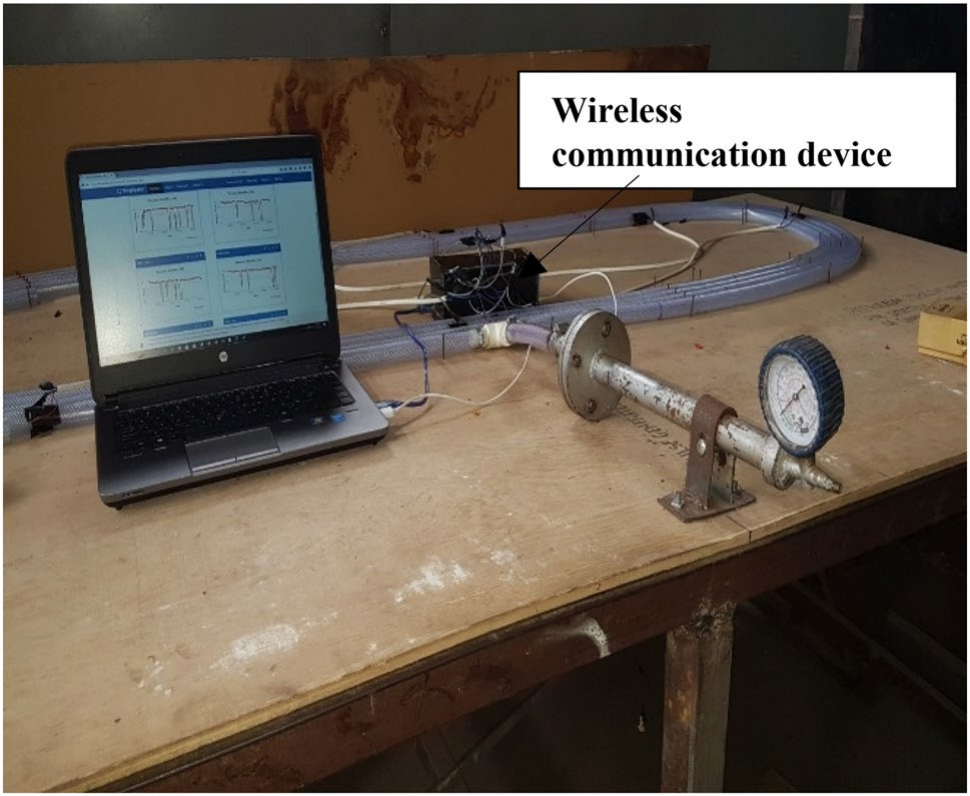
Experimental test rig 2 with wireless communication device

To validate the theory of event location as discussed above; two experimental flow loops as shown in Fig. 3 were built. This consisted of an air-filled PVC (Polyvinyl Chloride) pipe of total length 20.11 m and internal diameter of 20 mm along which pressure pulses propagated in both cases. A pressure pulse generator was used to introduce sharp-fronted pulses into the air-filled pipe in both cases too. Five piezoelectric sensors each were located at different positions along the PVC pipes. Sensor 1 was located at 2 m from one end; while sensor 2 was located at 5 m from one end and sensor 3 was located at 8 m from one end. Sensors 4 and 5 were located at 11 and 17 m from one end respectively. All sensors were connected to a single Pico Log data instrumentation system to capture and record the propagation of the pulses in the first experimental test rig. A second test rig was built with all the sensors connected to the wireless communication device. Both test rigs are shown in Figs. 4 and 5. The location of the event was 4.23 m from one end.

Sensor 1 was located at 2.24 m from the event location; sensor 2 was located at 0.68 m from the event location; sensor 3 was located at 3.68 m from the event location; sensor 4 was located at 6.68 m from the event location, and sensor 5 located at 12.684 m from the event location. Pressure pulses were generated at specific pressures severally and measurements made for each amount of pressure set in the pulse generator. The velocity of the propagated pulse was determined experimentally based on measured pressure at two sensors. These measured pulses were cross-correlated using the Matlab® software to estimate the delay between arrival times (t_delay_). The pulse propagation velocity was calculated as [24]:

3 $$ {\text{C}}_{p} = \frac{{ x_{ab} }}{{t_{delay ab} }} $$

where *x*_*ab*_ = distance between two selected sensors.

*t*_delay ab_ = time delay between arrival times at two selected sensors.

This calculation was done severally to examine the repeatability of pressure pulse velocity value. The average value of experimental pulse propagation velocity was determined from plots made graphically and compared to that obtained using Eq. 3 to validate its accuracy. These experimental values of velocity were then used in Eqs. 1 and 2 to calculate the various event locations. An average of these locations was calculated and compared against the actual measured event location to determine the accuracy of the method used. Calculation of event locations and pulse propagation velocity was done using data from the Pico Log data capturing device in the first experimental test rig only. The first test rig was built to validate the theory of damage location using pressure-induced pulses while the second test rig using the wireless communication device was built to validate the theory of pipeline monitoring using pressure-induced pulses on an IoT platform.

In the experiments using the first test rig, a voltage rating of 100 mV on the Pico Log data logger was used. The experiments using the pulse generator to create an impulsive event within the pipe were carried out repeatedly and the various wave spectra outputted after processing.

## Results and discussion

### Experimental results from test rig with data logger

**Fig. 6:**
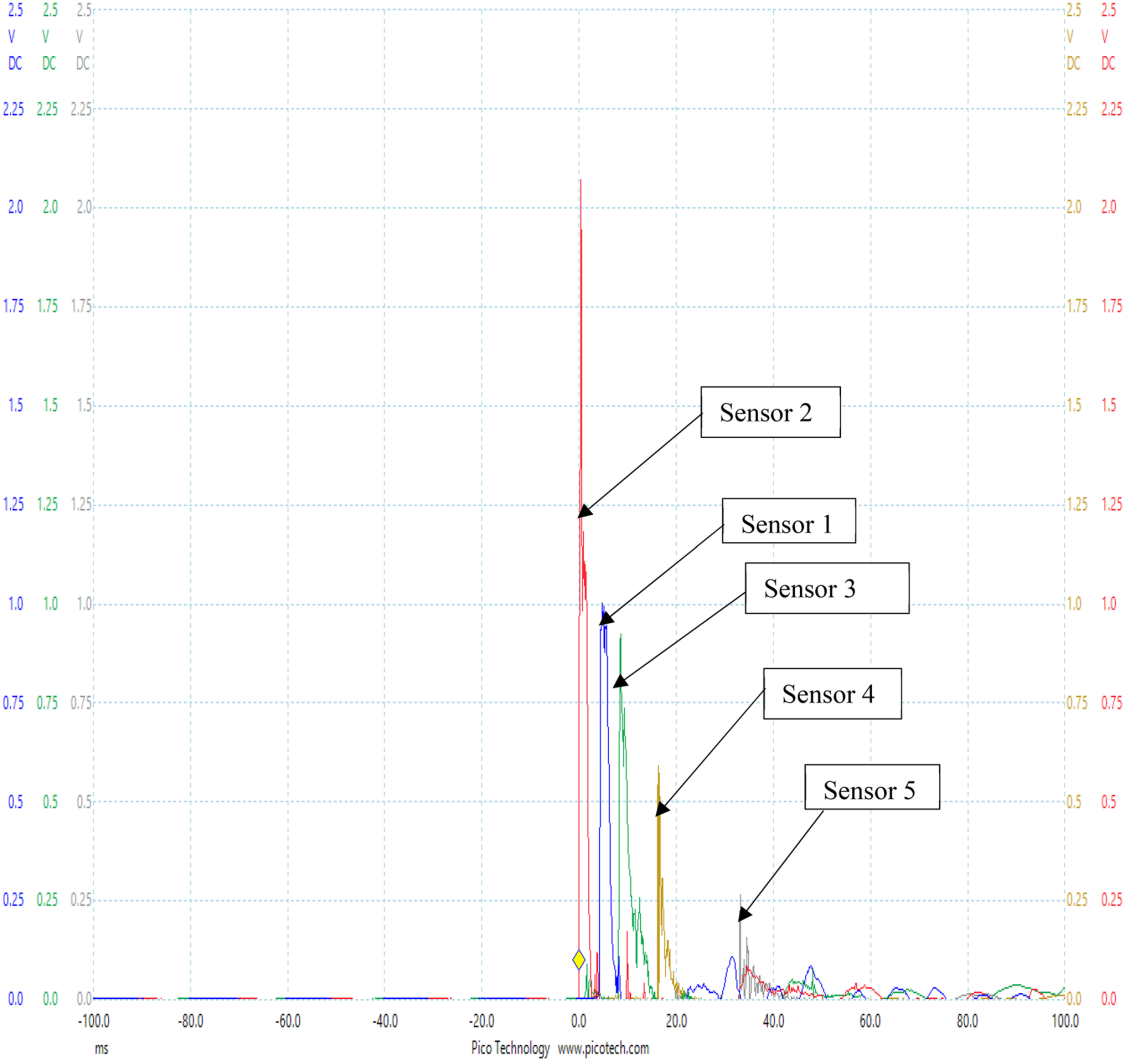
Pressure pulse measured at all five sensors of experimental rig at pressure of 1 bar with data logger

**Fig. 7:**
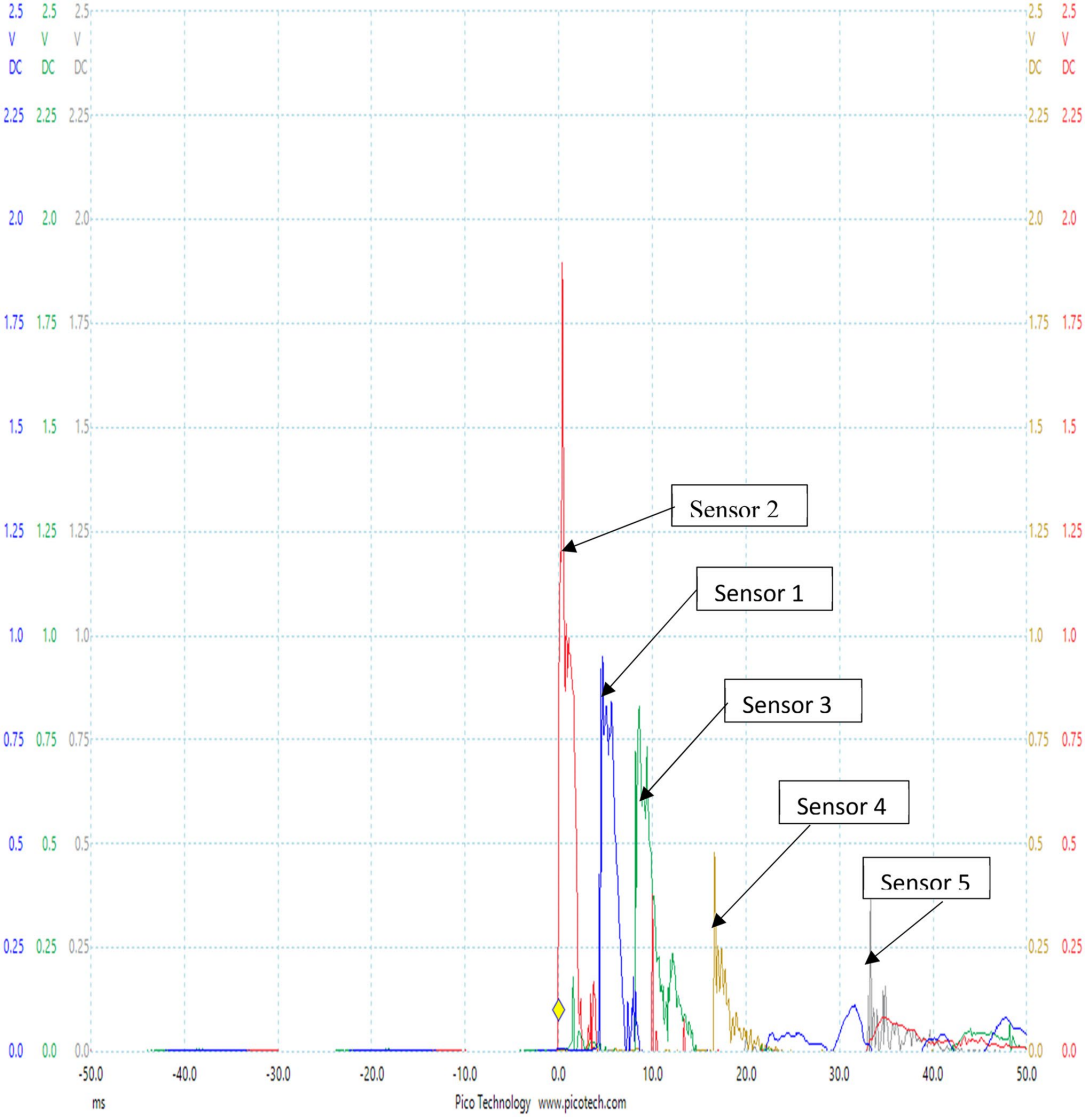
Pressure pulse measured at all five sensors of experimental rig at pressure of 0.8 bar with data logger

The experiments were carried out using the test rig shown in Fig. 4 with air as the transport fluid. Figures 6 and 7 shows the Pico Log representation of the pressure pulses captured at each of the five sensors located along the pipeline. A sampling rate of 13.16 Ks/s was used in measuring and recording the pulse signals at the four sensors.

Figures 6 and 7 show pressure pulses from two of the test results obtained at the five sensors located along the pipe of the rig at pressure readings of 1 bar and 0.8 bar respectively with air as the transport fluid. Located closest to the tee connection was sensor 2, meaning it was closest to the point of arrival of the pulse in the pipe. This is the point where the pulse enters the main pipe and therefore defines the event location. The pulse enters at this point and then sets off in both directions arriving first at sensor 2, followed by sensor 1, then sensor 3, then sensor 4, and finally sensor 5. This result confirmed the principle of delay in time arrivals of pulses at sensors that was adopted in this study. In the work, the arrival of pulses at sensors also varied according to their respective distances from the location of damage (the pulse generator). The sensor closest to the damage location gave the pulse with the highest amplitude and the sensor farthest from the damage location gave the pulse with the least amplitude. The shape and pattern of the pulses in both works were also similar. The best independent possible measurement of the event as it enters the pipe is sensor 2. This is because attenuation or distortion of the pulse propagating from the tee connection will be very little before it reaches sensor 2 as a result of its proximity to the tee connection. The remaining four sensors are located at different points along the pipe to aid the location of the event.

It is observed from Figs. 6 and 7 that the originally generated pressure pulse reflects back to the pulse generator. This reflection is observed to go back and forth from the pulse into the pipe. This negative pulse is not used in any of the experimental calculations.

### Velocity of pressure pulse propagation in static air

The measured pressure pulses at sensors 3 and 4 were used to determine the velocity at which the pressure pulse propagates through the pipe based on the configuration in Fig. 4 with air as the transport fluid. The Matlab® software was used to cross-correlate these measured pulses to estimate the delay between arrival times. The velocity of the propagation of the pressure pulse was computed based on Eq. 3.

The highest pressure pulse was generated with 1.0 bar in the pulse generator while the smallest pressure was generated with 0.2 bar. 50 measurements were made in total because 10 measurements each were made for each pressure rating in the pulse generator. An average of the time delay between pulse arrivals was obtained after cross correlation in Matlab® and the result of the average value used in Eq. 3 to obtain the velocity of pulse propagation as:

x_34_ = 3 m.

t_delay34_ = 0.008297 s

$$ {\text{C}}_{p air } = \frac{3}{{{ }0.00845}} $$

C_p air_ = 355 m/s.

The value of velocity obtained is more than the nominal velocity of sound in air which is 343 m/s [26]. The reason for this discrepancy was investigated and an increase in the temperature of the pulses in the pipe over the ambient was found to be responsible.

### Event location in static air

The Matlab® m-code language was used in the calculation of the event location of the test rig’s pipe with air as the transport fluid. The location of the real event is the point of entrance of the original pressure pulse into the main pipe and it was determined from measurements made at sensors 1 and 2. The tee connection is the actual source of the pulses and formed the basis of all calculations about the location of the event. Equation 2 was used to calculate the actual event location which is the event occurring at sensor 2. The equation was slightly modified because as opposed to Fig. 1, in the actual experimental test rig, the tee connection was located between sensors 1 and 2, but closest to sensor 2 (the event location). Therefore Eq. 2 was modified as:

4 $$ {\text{x}}_{{{\text{DE}}1}} = \frac{{\left( {t_{12} C_{p} { } + { }x_{21} } \right)}}{2} $$

Equation 4 (the distance of the event location from sensor 1) plus the known distance between sensor 2 and the tee connection (x _offset_) gave the location of the event. x_21_ is the known distance between sensor 1 and 2, while t_12_ is the measured time delay between pulse arrivals at sensors 1 and 2 which was obtained using the cross-correlation technique. With the aid of the written Matlab® code, a consistency in the computed event location estimates was observed. The computed estimates were in the range of between 4.243 and 4.246 m, and having a scatter of just 3 mm. The actual measured location of the event on the test rig was 4.23 m.

### Wireless processing and transmission of data

A wireless communication device was incorporated into the experimental test rig as shown in Fig. 5. The Arduino and Wi-Fi module were powered by a computer system through the use of two USB cables. This was so due to proximity to the computer system as the device could be powered by being connected to an electric source. The Wi-Fi module of the device was activated by the internet connection from an android phone. This device replaced the data logger that was used in the setup in Fig. 4 and in works by Junxiao et al. [6], Golmohamadi [8], and was an improvement on these works as the sensors captured the pressure pulses from the various damage events and the device transmitted these pulse data to the ThingSpeak analytics platform. This meant that monitoring of pipelines can be done in real time from any location in the world. Wire piezoelectric sensors were used in these experiments and the device was connected to the sensors via wires. Processing of these pulse data was done in real time on the ThingSpeak platform and the output of the measured pressure pulses was also displayed in real time.

**Fig. 8:**
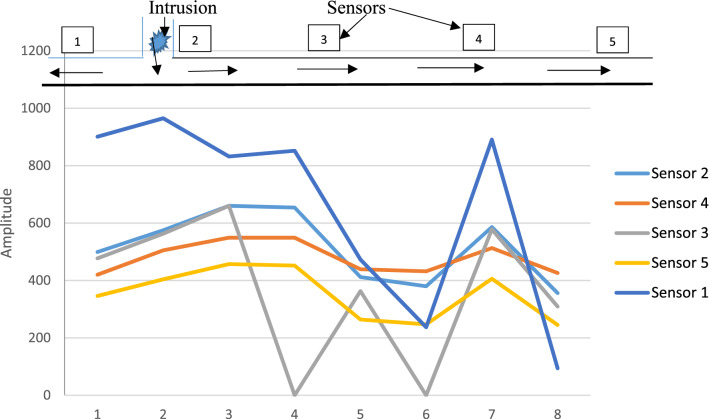
Matlab® representation of measured pressure pulse at all sensors using the wireless communication device for a 1 bar pressure reading in the pulse generator

The free student license of the ThingSpeak platform was used for this work and as a result, there was a 15 s delay in the transfer of pulse data to the platform. To achieve a one second transfer of pulse data on the platform, a professional license was required. Using a pulse generator, sharp fronted pulses were generated into the pipe and these captured pressure pulses were transmitted wirelessly via the wireless communication device to the ThingSpeak platform. Figure 8 shows the measured pressure pulses at sensor 1; sensor 2; sensor 3; sensor 4; and sensor 5 respectively for a pressure reading of 1 bar in the pulse generator.

The experimental setup was same as before with sensor 2 closest to the tee connection and taken as the damage location. On the ThingSpeak platform, sensor 1 readings were displayed on field 1 while sensor 2 readings were displayed on field 5. Sensor 3, 4 and 5 readings were displayed on fields 3, 2 and 5 respectively on the ThingSpeak platform.

Based on the sensor locations on the experimental set, a pressure pulse from the pulse generator would get to sensor 2 first, then to sensors 1, 3, 4, and 5 respectively. This was as a result of their respective distances from the pulse generator. The results of these experiments confirm the aforementioned and also the effectiveness of the wireless communication device.

For the case of pressure of 1.0 bar as in Fig. 8, the highest pulse amplitude value for all the five sensors was 908 mm, confirming that the pulse with a propagation velocity of 355 m/s also got to sensor 2 first. This was followed by an amplitude value of 509 mm, the second highest value for all five sensors confirming that the pulse also got to sensor 1 after sensor 2 in this case too. The third amplitude value was 487 mm; then 429 mm and finally 355 mm. This also goes to confirm that the pressure pulse eventually got to sensors 3, 4, 5 in that order respectively.

**Fig. 9:**
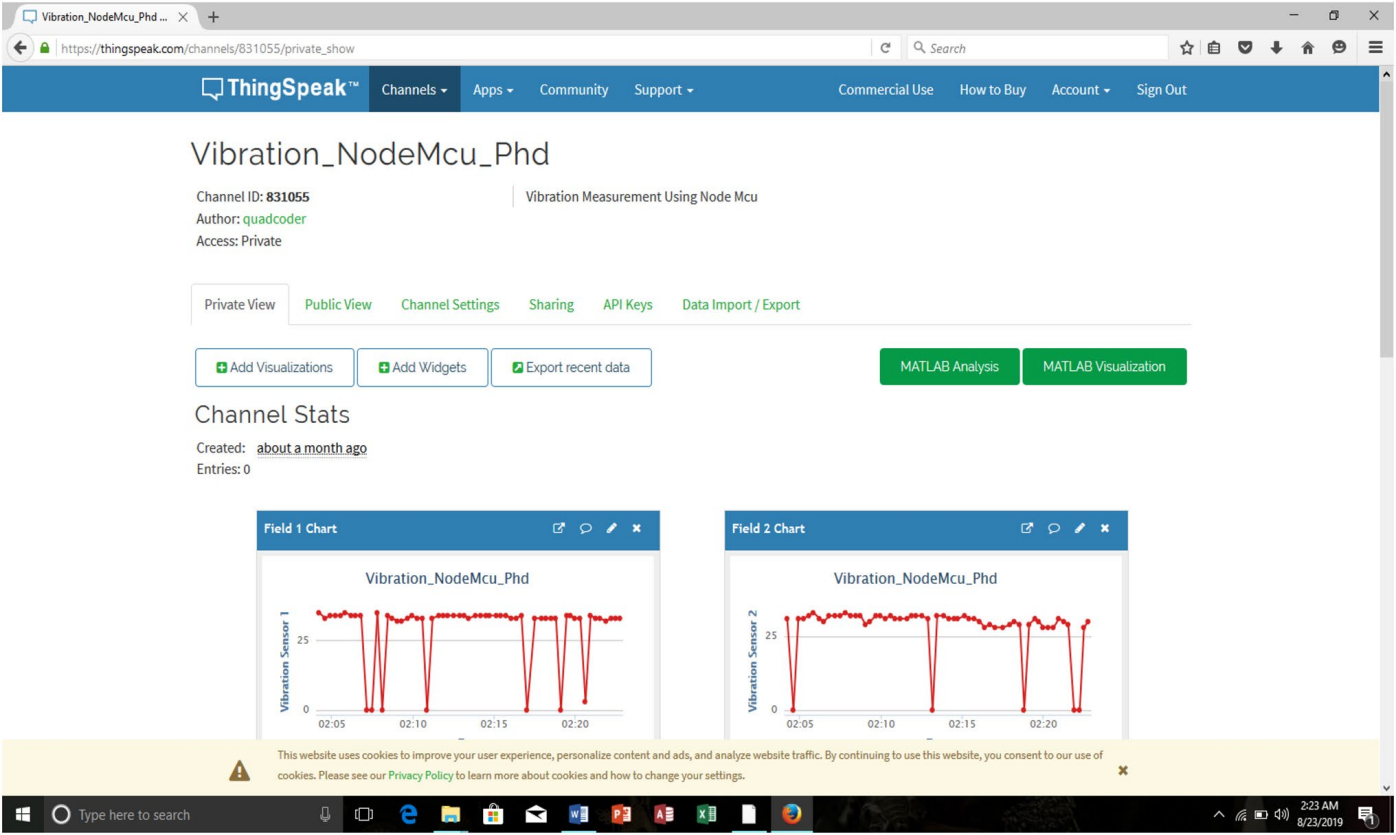
ThingSpeak analytics platform page showing measured pressure pulses from sensor channels 1 and 2 [23]

A total of 15 tests were repeated using five different pressure readings on the pulse generator and the wireless communication device, and the results were similar. A typical screen display of the ThingSpeak platform is as shown in Fig. 9.

## Conclusion

A wireless communication device was developed for transmission and processing of measured pressure pulses wirelessly to an IoT analytics platform (ThingSpeak) for real time monitoring. Tests rigs were built with static air as the transport fluid to validate the efficacy of the developed transmission device. Based on a previously developed method of locating the position of an intrusion event on a pipe that relies on the time delay between pulse arrivals at two sensors, a difference of 20 mm only was recorded between the computed and actual event locations when a data logger was used for capturing and transmission of sensor data. This was carried out as a way of validating the theory of pipeline monitoring based on pulse arrival times at sensors along the pipe. The wireless communication device was then used for capturing and transmission of data from sensors on the test rig’s pipe to the ThingSpeak platform. The results obtained were similar to those obtained using the data logger.

A low-cost pipeline monitoring system with the ability to perform real-time damage detection, location, and allows you to view the results of the measured pressure pulses in real time on a computer system or even on a smart phone from any location in the world was developed.
